# Sense of School Belonging as a Mediator of the Relationship between Witnessing Bullying and Internalizing Symptoms

**DOI:** 10.3390/ijerph21070873

**Published:** 2024-07-04

**Authors:** Diana M. Doumas, Aida Midgett

**Affiliations:** 1 Institute for the Study of Behavioral Health and Addiction, Boise State University, 1910 University Drive, Boise, ID 83725, USA; aidamidgett@boisestate.edu; 2 Department of Counselor Education, Boise State University, 1910 University Drive, Boise, ID 83725, USA

**Keywords:** witnessing bullying, sense of school belonging, internalizing symptoms, middle school

## Abstract

Bullying is a significant public health concern associated with mental health risks. Negative bullying outcomes extend beyond targets, with students who witness bullying reporting negative associated mental health consequences. Because bullying often occurs within the school setting, understanding the relationship between witnessing bullying and internalizing symptoms within the school environment can help shape school-based bullying prevention. The purpose of this study was to examine sense of school belonging as a mediator of the relationship between witnessing bullying and internalizing symptoms. We conducted two studies with middle school students (*N* = 130; *N* = 147) in which we used structural equation modeling (SEM) to test the mediational model. Results from Study 1 and Study 2 supported the mediational model, indicating that one explanation for the relationship between witnessing bullying and internalizing symptoms is that witnessing bullying negatively impacts students’ sense of school belonging, which in turn increases internalizing symptoms. Results from this study provide support for the importance of students’ perceptions of school climate in the development of internalizing symptoms related to witnessing bullying. Implications for school-based bullying prevention programs are discussed.

## 1. Introduction

School bullying is a significant public health concern in the United States, with national survey data indicating 22.2% of students aged 12–18 report being a target of bullying [1]. Bullying peaks in middle school, with the highest rates reported by sixth grade students (29.5%) [2]. A large body of research indicates that bullying victimization is associated with a wide range of negative outcomes, including poor general health, anxiety and post-traumatic stress, depressive symptoms, non-suicidal self-injury, suicidal ideation, and suicide attempts [3]. There is also evidence to suggest a causal relationship between bullying victimization and anxiety and depression [4]. Bullying victimization is also associated with negative academic outcomes, increased peer rejection, and poorer school connectedness, both in the short term and nearly a decade after victimization [5]. Further, the negative effects of childhood bullying extend to adulthood, with adults who experienced bullying victimization in childhood reporting higher rates of legal problems, violence, and substance misuse than those who were not victimized [6]. 

### 1.1. Witnessing Bullying

Research indicates that 80% of students report witnessing bullying at school [7]. Similar to students who experience bullying victimization, the negative consequences associated with bullying extend to students who witness other students being bullied. For example, witnessing school bullying is associated with depressive symptoms [8,9,10], suicide risk [11], anxiety [10], and social anxiety [9], even when controlling for the effects of bullying victimization. Further, witnessing cyberbullying is associated with depression and anxiety [12,13,14], and somatic symptoms [12]. Further, researchers have found that witnessing both school bullying and cyberbullying is associated with higher levels of depressive symptoms compared to witnessing either school bullying or cyberbullying [15]. Additionally, researchers have found that witnessing school bullying [11] and witnessing cyberbullying [16] are both related to suicidal ideation and that this relationship is mediated by internalizing symptoms. Thus, it is important to understand factors that may contribute to internalizing symptoms among students who witness bullying.

### 1.2. Sense of School Belonging 

Schools serve as one of the primary environments for socio-emotional development among youth [17]. According to socio-ecological theory, behavior is influenced by the complex interaction of individual and environmental factors [18]. Within the socio-ecological model, bullying can be viewed within the context of the school system, including school climate. Of the many components that make up school climate, sense of school belonging has been extensively studied in regard to the need for relatedness [19]. Sense of school belonging can be conceptualized as both having positive relationships with peers and adults at school and feeling a sense of safety and belonging in the school environment [20]. Researchers have found that witnessing bullying is related to feelings of isolation, disconnectedness, and being cut off by their peers, particularly when they do not intervene in the bullying situation [21]. Among adolescents, a sense of school belonging is also inversely related to depressive symptoms [22]. Thus, one of the reasons witnessing bullying may be related to depression and anxiety may be due to low levels of sense of school belonging. 

### 1.3. The Current Study

**Figure 1:**
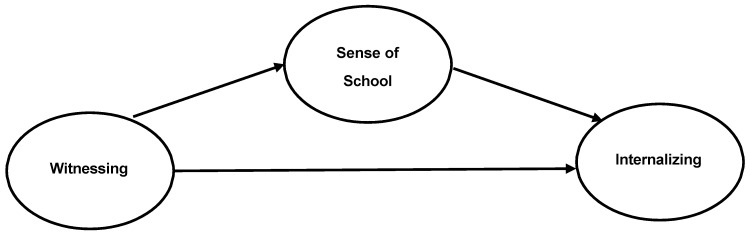
Conceptual Model.

Research indicates that witnessing bullying is associated with internalizing symptoms among middle school students. How witnessing bullying is related to internalizing symptoms, however, is unclear. Given the relationship between sense of school belonging and feelings of isolation, disconnectedness [21], and depressive symptoms [22], it is possible that sense of school belonging may mediate the relationship between witnessing school bullying and internalizing symptoms. Thus, the aim of this study is to extend the literature by examining sense of school belonging as a mediator of the relationship between witnessing bullying behavior and internalizing symptoms (see Figure 1). To achieve this aim, we examined these relationships in two samples of middle school students, one with sixth through eighth graders (*N* = 130) and one with only sixth graders (*N* = 147), using different measures for internalizing symptoms in order to strengthen the findings through replication and provide increased generalizability.

We hypothesized that witnessing bullying would decrease a sense of school belonging, which, in turn, would increase internalizing symptoms. Specifically, in Study 1, we hypothesized that sense of school belonging would mediate the relationship between witnessing bullying and internalizing symptoms (e.g., depressive symptoms, anxiety symptoms, and somatic symptoms) among sixth through eighth grade school students. In Study 2, we hypothesized that sense of school belonging would mediate the relationship between witnessing bullying and internalizing symptoms (i.e., depressive symptoms and social anxiety) among sixth grade students. We used structural equation modeling to test both the direct and indirect effects (e.g., mediation) in both studies.

## 2. Study 1 

### 2.1. Methods

#### 2.1.1. Participants and Procedures

We recruited participants from one public middle school in the Northwest of the United States in 2018. We randomly selected 360 students using a stratified proportionate sampling procedure. Participants included 130 students (57.4% female; 42.6% male), with 36.2% in sixth grade, 33.1% in seventh grade, and 30.8% in eighth grade. Participants ranged in age from 11 to 15 years old (*M* = 12.50 and *SD* = 1.00). Racial/ethnic composition included 58.5% White, 36.9% Hispanic, 1.5% Asian/Pacific Islander, 0.8% African-American, 0.8% Asian-American, and 1.5% Other. 

The research team worked with the school counselor to implement research procedures. The school sent three mailings to the parents/guardians of selected students, including a pre-notification, consent, and reminder letter explaining the research project. Materials were provided in both Spanish and English. Parents/guardians were asked to return the consent form in a project-addressed stamped envelope if they agreed to their student’s participation. Additionally, the school counselor and a team member hand-delivered the letter and consent form to students to take home to their parents/guardians. Parental/guardian consent was obtained from 142 students (39.4%). Of these students, 130 provided assent for a final response rate of 36.1%. Students completed data collection during class time. Study incentives included a pizza party. All study procedures were approved by the University Internal Review Board and by the School District. 

#### 2.1.2. Measures

##### Bullying Victimization

We used three measures to assess bullying victimization. First, we used the Olweus Bullying Questionnaire [23] to measure the frequency of witnessing bullying in the past 30 days. As part of the questionnaire, participants were provided with a definition of bullying that includes specific examples of different types of bullying (i.e., physical, verbal, and relational bullying). Further, the definition clarifies what bullying is (e.g., actions that happen repeatedly, making it difficult for students to defend themselves, or being teased repeatedly in a mean or hurtful way) and what bullying is not (e.g., teasing in a friendly way or two students of equal power arguing or fighting). Students are then asked to complete the following item: “How often have you seen another student being bullied at school in the past 30 days?” to measure witnessing bullying. Items are rated on a 5-point Likert Scale ranging from 0 (*I Have Not*) to 4 (*Several Times a Week*). Second, we assessed the frequency of witnessing bullying using questions from prior research [15]. Students were asked, “How often have you seen the following types of bullying in the past month?” The types of bullying listed were physical, verbal, relational, and cyberbullying. Examples of each type of bullying were provided. The items are rated on a 5-point Likert Scale ranging from 0 (*Never*) to 4 (*Several Times a Day*). We summed the four items to obtain the frequency of witnessing bullying (α = 0.72). Finally, we assessed witnessing bullying by asking participants, “Have you seen bullying at school in the past month?” with response choices *Yes* and *No*. Researchers have utilized this item to measure witnessing bullying as a dichotomous variable among middle school students [24,25].

##### Sense of School Belonging

The Psychological Sense of School Membership (PSSM) [26] was used to measure a sense of school belonging. The PSSM is an 18-item self-report inventory that measures students’ perceptions of belonging to their school. Example items include: “I feel like a real part of my school”, “People notice when I am good at something”, The teachers here respect me”, “People at this school are friendly to me”, and “Other students here like me the way I am”. Items are rated on a 5-point Likert Scale ranging from 1 (*Not True at All*) to 5 (*Completely True*), with five of the items reverse scored. Overall, the PSSM has well established concurrent and predictive validity, as well as supported factor structures [27]. Researchers have also reported test-retest reliability for the PSSM over a 4-week period of 0.78 [28] and 0.56–0.60 over a 12-month period [22]. Reported coefficient alphas range from 0.78 to 0.95 [27]. For this sample, Cronbach’s alpha was 0.79.

##### Depressive Symptoms

We used the 12-item Depression Scale of the Behavioral Assessment System for Children, Third Edition Self Report of Personality-Adolescent Form (BASC-3 SRP-A) [29] to measure symptoms of depression. Five items are rated on a dichotomous scale: 0 (*True*) or 2 (*False*). Example items include: “I do not seem to do anything right”, “I just do not care anymore”, and “I used to be happier”. Seven items are rated on a 4-point Likert Scale ranging from 0 (*Never*) to 3 (*Almost Always*). Examples include: “I feel depressed”, “I feel life is not worth living”, and “I feel like I have no friends”. We obtained a total scale score through the BASC-3 SRP-A hand-scoring worksheet [29]. The BASC-3 SRP-A Depression Scale has reliability coefficient alphas ranging in the 0.80s and evidence of construct validity [29]. For this sample, Cronbach’s alpha was 0.92. 

##### Anxiety Symptoms

We used the 13-item Anxiety Scale of BASC-3 SRP-A [29] to measure anxiety symptoms. Three items are rated on a dichotomous scale of 0 (*True*) or 2 (*False*). Example items include: “I can never seem to relax”, “I often worry about something bad happening to me”, and “I worry a lot of the time”. Ten items are rated on a 4-point Likert Scale ranging from 0 (*Never*) to 3 (*Almost Always*). Examples include: “I feel anxious”, “I get so nervous I cannot breathe”, and “I worry when I go to bed at night”. The total scale score was obtained through the BASC-3 SRP-A hand-scoring worksheet [29]. The BASC-3 SRP-A Anxiety scale has reliability coefficient alphas ranging in the 0.80s and evidence of construct validity [29]. For this sample, Cronbach’s alpha was 0.88.

##### Somatic Symptoms

We used the 9-item Somatic Complains Subscale of the Child Behavioral Checklist (CBCL) [30]. Example items include: “I feel dizzy or lightheaded”, “I have headaches”, and “I have stomachaches”. Items are rated on a 4-point Likert Scale ranging from 0 (*Not True*) to 2 (*Very True or Often True*). Items are summed to create a total subscale score. The CBCL has established reliability and validity, with the internal consistency of the Somatic Complaints Subscale reported at 0.78 [30]. For this sample, Cronbach’s alpha was 0.73.

#### 2.1.3. Data Analysis

Prior to analysis, variables were examined for normality. We also assessed for multicollinearity using the VIF rule-of-thumb of VIF < 10 [31]. We examined the data for missing values and imputed missing data using maximum likelihood (ML) estimation [32]. SEM analyses were conducted using AMOS 29.0 (IBM, Chicago, United States) with the maximum likelihood estimation method to evaluate model fit and examine the direct and indirect relationship between witnessing bullying, sense of school belonging, and internalizing symptoms. We selected SEM as this method of analysis allows for the examination of direct and indirect paths between observed variables [33]. We used three goodness of fit indices to assess model fit: chi-square, the comparative fit index (CFI), and the root mean square error of approximation (RMSEA). Good model fit is demonstrated when the model chi-square is not significant, the CFI value is 0.95 or greater, and the RMSEA value is 0.07 or less [34]. We used an alpha level of *p* < 0.05 to determine statistical significance.

To test for mediation, we used the joint significance test of indirect paths [35] from the predictor (i.e., witnessing bullying) to the mediator (i.e., sense of school belonging), and from the mediator to the outcome (i.e., internalizing symptoms). We used bias-corrected bootstrapping, in which indirect effects are estimated from multiple resampling of the data set. We used confidence intervals to provide a range of estimates for the mediated effect [36]. Confidence intervals that do not include zero indicate the significance of the indirect effect [37]. We tested the indirect effect with 5000 bootstrap samples and a 95% confidence interval (CI). 

### 2.2. Results 

**Figure 2:**
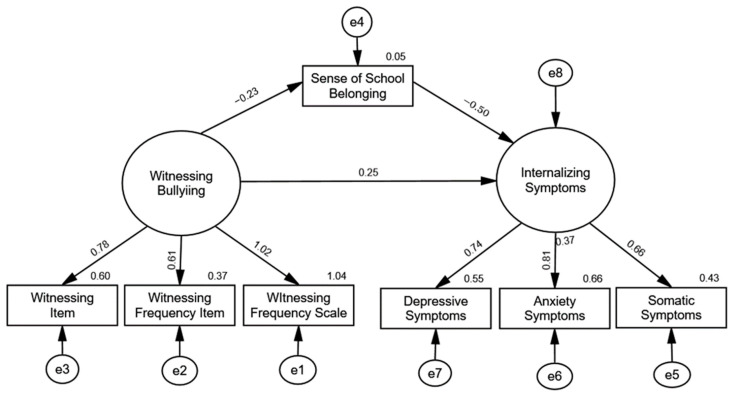
Study 1 SEM Mediation Model. Note. Latent variables are indicated with ovals, and observed variables are indicated with rectangles.

Correlations for the variables in the model are presented in Table 1. The mediational model results are depicted in Figure 2. Examination of the global fit statistics indicated that the model was a good fit for the data. The overall chi-square test of the model was statistically nonsignificant, χ^2^(12) = 20.33, *p* = 0.06, the CFI was 0.98, and the RMSEA was 0.07. As seen in Table 2, examination of the path coefficients revealed that witnessing bullying was significantly related to lower levels of sense of school belonging, β = −0.23, *p* = 0.006, sense of school belonging was significantly related to higher levels of internalizing symptoms, β = −0.50, *p* = 0.001, and witnessing bullying was significantly related to higher levels of internalizing symptoms through the mediated effect of decreased levels of sense of school belonging, β = 0.12, *p* = 0.004, 95% CI [0.03, 0.22]. These results offer evidence that a sense of school belonging mediated the relationship between witnessing bullying and internalizing symptoms.

## 3. Study 2 

### 3.1. Methods

#### 3.1.1. Participants and Procedures

We recruited all sixth grade students (*N* = 354) from one public middle school in the Northwest region of the United States in 2020. Participants included 147 students (59.2% female; 38.8% male; 2.0% missing). Participant ages ranged from 11 to 12 years old (*M* = 11.45, *SD* = 0.50). Racial/ethnic composition included 52.1% White, 30.7% Hispanic, 2.2% African American, 1.4% Asian American, and 13.6% Multiracial or Other. 

The research team worked with the school counselor to implement research procedures. The school sent information about the study and an informed consent form to parents/guardians for them to sign if they agreed to their child’s participation. The school counselor also visited all sixth grade classrooms and hand-delivered consent forms to students to take home to their parents/guardians. Students who returned a completed informed consent form received one piece of candy from their teacher or school counselor to incentivize their participation. Immediately prior to data collection, students with parental/guardian consent provided assent before completing the study survey. A total of 147 (41.5%) students returned a signed parent/guardian consent form and agreed to participate. Students completed surveys during class time. 

#### 3.1.2. Measures

##### Bullying Victimization

We used the same three measures to assess bullying victimization as in Study 1. For the frequency of witnessing bullying scale [15], for this sample, Cronbach’s alpha was 0.70. 

##### Sense of School Belonging

We used the same measure to assess a sense of school belonging as in Study 1. For this sample, Cronbach’s alpha was 0.71.

##### Depressive Symptoms

We used the 20-item Center for Epidemiological Studies Depression Scale for Children (CES-DC) [38] to measure depressive symptoms. Example items include “I was bothered by things that usually do not bother me”, “I felt like I was too tired to do things”, and “I felt sad”. All items are summed to obtain a total score. Items are rated on a 4-point Likert Scale ranging from 0 (*Not at All*) to 3 (*A Lot*). Researchers have demonstrated construct validity (Weissmann et al. 1980) [31], good to moderate test-retest reliability [39], and good internal reliability (α = 0.89) [40]. For this sample, Cronbach’s alpha was 0.90.

##### Social Anxiety

We used the 22-item Social Anxiety Scale for Adolescents (SAS-A) [41] to measure social anxiety. For this study, we used a 9-item scale comprised of items from the Social Avoidance and Distress Scale—General (SAD-General) and Fear of Negative Evaluation Scale (FNE) [42]. Example items include “I am worried about what others say about me”, “I stay quiet when I am in a group of people”, and “I am afraid others would not like me”. Items are rated on a 5-point Likert Scale ranging from 0 (*Not at All*) to 4 (*All the Time*). Items are summed for a total score on each scale. Researchers have also demonstrated moderate test-retest reliability (0.62 for SAD-General and 0.55 for FNE over a 12-month period) [43] and adequate to good internal reliability with Cronbach α coefficients ranging from 0.76 for SAD-General and 0.91 for FNE [44]. For this sample, Cronbach’s alpha was 0.94.

#### 3.1.3. Data Analysis

We followed the same procedures as in Study 1 for preliminary analyses, including the examination of variables for normality, assessing for multicollinearity, and imputing missing data. SEM analyses were conducted using AMOS 29.0 with the maximum likelihood estimation method to evaluate model fit and examine the direct and indirect relationship between witnessing bullying, sense of school belonging, and internalizing symptoms. We used the three goodness of fit indices detailed in Study 1 (i.e., chi-square, CFI, and RMSEA. We used an alpha level of *p* < 0.05 to determine statistical significance. Following the procedures in Study 1, we used the joint significance test of indirect paths [35] and confidence intervals to provide a range of estimates for the mediated effect [36]. We tested the indirect effect with 5000 bootstrap samples and a 95% confidence interval (CI). 

### 3.2. Results 

**Figure 3:**
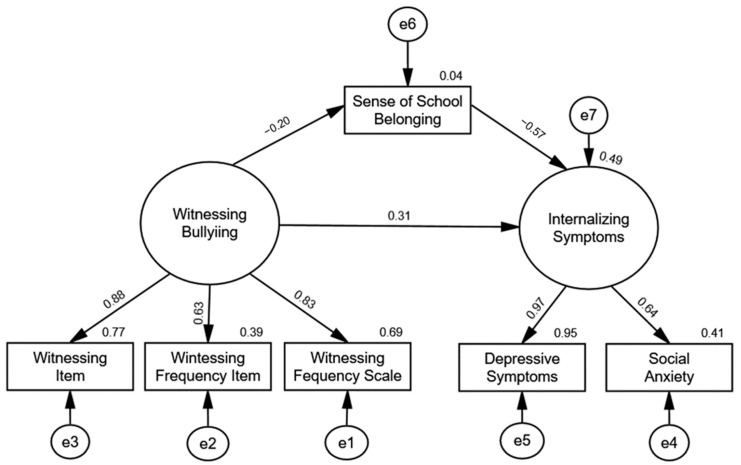
Study 2 SEM Mediation Model. Note. Latent variables are indicated with ovals, and observed variables are indicated with rectangles.

Correlations for the variables in the model are presented in Table 3. The mediational model results are depicted in Figure 3. Examination of the global fit statistics indicated that the model was a good fit for the data. The overall chi-square test of the model was statistically nonsignificant, χ^2^(7) = 9.04, *p* = 0.25, the CFI was 0.99, and the RMSEA was 0.05. As seen in Table 4, examination of the path coefficients revealed that witnessing bullying was significantly related to lower levels of sense of school belonging, β = −0.20, *p* = 0.02, sense of school belonging was significantly related to higher levels of internalizing symptoms, β = −0.57, *p* = 0.001, and witnessing bullying was significantly related to higher levels of internalizing symptoms through the mediated effect of decreased levels of sense of school belonging, β = 0.11, *p* = 0.02, 95% CI [0.03, 0.40]. These results offer evidence that a sense of school belonging mediated the relationship between witnessing bullying and internalizing symptoms.

## 4. Discussion

The purpose of this study was to examine sense of school belonging as a mediator of the relationship between witnessing bullying and internalizing symptoms among middle school students. Although prior research indicates witnessing bullying [8,9,10,12,13,14] is associated with internalizing symptoms, to our knowledge, this is the first study to examine a sense of school belonging as an explanatory factor in this relationship. Because approximately 80% of students report witnessing bullying [7] and are at risk for the associated negative mental health outcomes, it is important to identify factors that impact the relationship between witnessing bullying and internalizing symptoms. To achieve our study aims, we examined the mediational model with two distinct samples. Study 1 included middle school students in sixth through eighth grade, and Study 2 focused exclusively on sixth grade students to specifically examine these relationships at the grade level when bullying peaks. Overall, results from both studies supported the mediational model, demonstrating that a sense of school belonging mediated the relationship between witnessing bullying and internalizing symptoms. These findings provide important information related to the role of the school environment in bullying prevention and intervention programs for middle school students. 

Results from this study indicated that witnessing bullying was related to higher levels of internalizing symptoms. This finding is consistent with the growing body of research that indicates witnessing both school bullying [8,9,10] and cyberbullying [12,13,14] are related to internalizing symptoms, including depressive symptoms, anxiety symptoms, social anxiety, and somatic symptoms. Additionally, results demonstrated that witnessing school bullying is related to lower levels of a sense of school belonging. This finding adds to the literature supporting the relationship between witnessing bullying and feelings of isolation, disconnectedness, and being cut off by peers [21]. Further, our finding that lower levels of sense of school belonging negatively impact internalizing symptoms parallels prior research indicating an inverse relationship between sense of school belonging and depressive symptoms [22]. Findings from the current study extend the literature by demonstrating that a sense of school belonging mediates the relationship between witnessing bullying and internalizing symptoms. When students witness bullying, their sense of belonging to their school is negatively impacted, which in turn contributes to higher levels of internalizing symptoms, including depression, anxiety, and somatic symptoms. 

Findings from this study are consistent with socio-ecological theory, which highlights the complex interaction of individual (e.g., student) and environmental (e.g., school) factors [18]. One explanation for the importance of a sense of school belonging in the relationship between witnessing bullying and internalizing symptoms may be related to how students behave when they witness bullying. For example, some research suggests that when students who witness bullying intervene, they report higher levels of social acceptance and lower levels of social rejection [45]. In contrast, other research indicates that defending behavior is related to higher levels of internalizing symptoms [7,8,46]. Students who witness bullying may feel peer pressure to not intervene due to pro-bullying school norms or be concerned they will become the targets of bullying themselves [7]. Further, students who intervene in schools with pro-bullying norms may end up being socially isolated [7]. Although not assessed in the current study, the choice of whether or not to intervene in bullying situations, as well as school bullying norms, may be important factors to consider in understanding how witnessing bullying impacts a sense of school belonging and, in turn, internalizing symptoms. 

### 4.1. Limitations and Directions for Future Research

While this study contributes to our understanding of how witnessing bullying influences internalizing symptoms, certain limitations should be noted. First, although our study is comprised of two samples, the samples in Study 1 and Study 2 were both relatively small and were recruited from the same region in the Northwest, limiting the generalizability of the results. Future research with larger samples is needed to replicate these findings. Next, information was obtained through student self-report, potentially leading to biased or distorted reporting. Research, however, suggests that children are able to provide useful information when asked questions using Likert-type scales [47]. Further, this study was limited to studying one mediator (i.e., sense of school belonging) as an indicator of school climate. Future research should include defending behavior as an additional mediator to provide important information to further explain the link between witnessing bullying and a sense of school belonging. Further, other measures of school climate, including school bullying norms, could be included as moderators, as the mediational relationships may be different depending upon the school climate as it relates to bullying norms. Finally, examining demographic variables (e.g., race/ethnicity, gender, and age) as moderators of the mediational relationship was beyond the scope of this study and could be addressed in future research with larger samples.

### 4.2. Implications for School-Based Bullying Prevention

This study has important implications for school-based bullying prevention. First, it is important for school personnel, including school mental health professionals and teachers, to understand that the negative impact of bullying extends beyond the targets of bullying to students who witness bullying. Thus, it is important to focus on students who witness bullying when planning bullying prevention. Findings from this study suggest that an increased sense of school belonging may reduce the negative impact of witnessing bullying on internalizing symptoms. Witnessing bullying is related to feelings of isolation and disconnectedness from peers, particularly when they do not intervene in the bullying situation [21]. Thus, one way to increase a sense of school belonging for students who witness bullying is to provide bullying bystander training. Research indicates that when students are trained to intervene in bullying situations using specific pro-social skills, their sense of school belonging increases [48], and their internalizing symptoms decrease [24,48,49,50]. Further, because witnessing bullying is related to feelings of isolation and disconnectedness for students who do not intervene [21], providing bystander training that supports students with the skills to intervene in bullying situations may be particularly helpful. Thus, school personnel can increase their sense of school belonging in the context of witnessing bullying by providing bullying bystander training, including the use of pro-social skills to intervene in bullying situations. 

Additionally, student perceptions of the school climate affect the negative emotional outcomes associated with witnessing bullying. Establishing anti-bullying norms may be particularly important as they promote a school environment in which bullying is considered unacceptable. When school personnel exhibit anti-bullying behavior, they establish norms that promote a positive school climate and promote students’ sense of connectedness and feelings of being cared for by adults in the school environment [51]. Thus, findings from the current study highlight the importance of bullying prevention programs that include education and skills training to support anti-bullying norms and behaviors. Further, research specific to training teachers on how to support students who witness bullying indicates that post-training, teachers reported an increase in knowledge about bullying, confidence to support students when they intervene in bullying situations, confidence and comfort in managing bullying, and self-efficacy related to managing bullying [52]. School mental health professionals can provide training for teachers to provide the education and skills needed to support students to intervene when they witness bullying. Establishing anti-bullying norms and equipping school personnel with the skills they need to intervene in bullying may be an important component of bullying prevention, not only to reduce bullying itself but also to support students who witness bullying.

## 5. Conclusions

Witnessing bullying is associated with mental health risks, including internalizing symptoms. The results of this study point to the importance of a sense of school belonging as a mediator of the relationship between witnessing bullying and internalizing symptoms among middle school students. Findings from this study highlight the importance of students’ perceptions of their connection to the school environment in the development of internalizing symptoms related to witnessing bullying. Therefore, it is important for school-based bullying prevention programs to address both individual factors (e.g., student skills to intervene) and environmental factors (e.g., school bullying norms) through student bullying bystander training, as well as training school personnel to support students to intervene and to create a positive school climate. 

## Figures and Tables

**Table 1:** Bivariate Correlations for Variables in the Model.

Measure	1	2	3	4	5	6	7
1. Witnessing Bullying Item	__						
2. Witnessing Bullying Frequency Item	0.44 **	__					
3. Witnessing Bullying Frequency Scale	0.79 **	0.60 **	__				
4. Sense of School Belonging	−0.05	−0.28 **	−0.08	__			
5. Depressive Symptoms	0.25 **	0.17	0.26 **	−0.14	__		
6. Anxiety Symptoms	0.29 **	0.26 **	0.35 **	−0.10	0.62 **	__	
7. Somatic Symptoms	0.15	0.13	0.22 **	−0.18 *	0.44 **	0.56 **	__

* *p* < 0.05, ** *p* < 0.01.

**Table 2:** Study 1 Results of Mediation Analyses.

Effect	E	SE	Lower 95% CI	Upper 95% CI	*p*-Value
Witnessing Bullying Effect on Sense of School Belonging	−0.63	−0.23	−0.38	−0.07	0.006
Sense of School Belonging Effect on Internalizing Symptoms	−0.08	−0.50	−0.66	−0.29	0.001
Witnessing Bullying Effect on Bullying Victimization	0.01	0.25	0.06	0.41	0.01
Indirect Effect	0.05	0.12	0.03	0.22	0.004

Note. E = Unstandardized Coefficient; SE = Standardized Coefficient; CI = Confidence Interval.

**Table 3:** Bivariate Correlations for Variables in the Model.

Measure	1	2	3	4	5	6
1. Witnessing Bullying Item	__					
2. Witnessing Bullying Frequency Item	0.56 **	__				
3. Witnessing Bullying Frequency Scale	0.17 **	0.13	__			
4. Sense of School Belonging	−0.16	−0.19 *	−0.21 **	__		
5. Depressive Symptoms	0.35 **	0.29 **	0.06	−0.61 **	__	
6. Social Anxiety	0.15	0.16	0.14	−0.46 **	0.63 **	__

* *p* < 0.05, ** *p* < 0.01.

**Table 4:** Study 2 Results of Mediation Analyses.

Effect	E	SE	Lower 95% CI	Upper 95% CI	*p*-Value
Witnessing Bullying Effect on Sense of School Belonging	−0.80	−0.20	−0.40	−0.02	0.02
Sense of School Belonging Effect on Internalizing Symptoms	−0.21	−0.57	−0.73	−0.36	0.001
Witnessing Bullying Effect on Bullying Victimization	0.47	0.31	0.16	0.45	0.001
Indirect Effect	0.17	0.11	0.03	0.40	0.02

Note. E = Unstandardized Coefficient; SE = Standardized Coefficient; CI = Confidence Interval.

## Data Availability

Data are not available due to ethical restrictions.
